# Duration of antibody responses following severe acute respiratory syndrome coronavirus 2 infection

**DOI:** 10.1007/s11845-021-02860-4

**Published:** 2022-01-04

**Authors:** Yao Jiang, Xiuqi Wei, Hui Wang, Guiling Li

**Affiliations:** Cancer Center, Union Hospital, Tongji Medical College, Huazhong University of Science and Technology, Wuhan, 430022 China

**Keywords:** COVID-19, Humoral immunity, Immunoglobulin G, Immunoglobulin M, SARS-CoV-2

## Abstract

**Background:**

Little is known on the duration of anti-severe acute respiratory syndrome coronavirus 2 (SARS-CoV-2) immunoglobulin G (IgG) antibodies in patients following SARS-CoV-2 infection.

**Aims:**

We aimed to determine the duration of the immunoglobulin G (IgG) and M (IgM) antibody responses following SARS-CoV-2 infection and to evaluate the risk factors for a short duration of anti-SARS-CoV-2 IgG.

**Methods:**

We measured antibody responses in 94 patients who had recovered from SARS-CoV-2 infection. The chi-square test and multivariable logistic regression analysis were used to identify risk factors for a short duration (< 6 months) of anti-SARS-CoV-2 IgG.

**Results:**

IgG antibodies were detectable in all patients until 4 months; 19 (21.8%) convalescent patients reverted to IgG negative 4–6 months after symptom onset. IgM antibodies decreased significantly to 5.7% at 4–6 months after symptom onset. Patient characteristics were not associated with a short duration of detectable IgG.

**Conclusions:**

A substantial fraction of convalescents may exhibit a transient IgG response following SARS-CoV-2 infection. Our findings suggest that patients who have recovered from SARS-CoV-2 infection should also be vaccinated if their anti-SARS-CoV-2 IgG antibodies are undetectable.

## Introduction

Coronavirus disease 2019 (COVID-19), which is caused by severe acute respiratory syndrome coronavirus 2 (SARS-CoV-2) infection, was first detected in December 2019 and has since become a global pandemic [1]. As of 19 December 2020, 74,299,042 cases of COVID-19 have been confirmed, and 1,669,982 related deaths were reported. These cases have led to significant challenges for health care systems.

Several published studies have described antibody responses to SARS-CoV-2 in COVID-19 patients [2–8]. Zhao et al. demonstrated that the median seroconversion time for IgM and IgG were day 12 and day 14, respectively [9]. However, data regarding the duration of anti-SARS-CoV-2 antibodies are limited. Thus, we performed this study of convalescent COVID-19 patients to evaluate the duration of antibody responses after SARS-CoV-2 infection, and factors associated with the duration.

## Methods

### Study participants

The Cancer Center, Union Hospital, which is affiliated with the Tongji Medical College of Huazhong University of Science and Technology, was designated as a hospital of admission for COVID-19 patients in Wuhan, China, during the SARS-CoV-2 pandemic. This study included patients admitted to the Cancer Center with laboratory-confirmed SARS-CoV-2 infection. They had either positive reverse transcription polymerase chain reaction tests for SARS-CoV-2 RNA or positive anti-SARS-CoV-2 antibody tests. All of them were symptomatic with clinical symptoms including fever, cough, fatigue, dyspnea, myalgia, and diarrhea and had chest radiographic findings of pneumonia.

### Collection of specimens and information

This study was approved by the Union Hospital of Huazhong University of Science and Technology ethics committee (20200191), which waived the requirement for informed consent due to the urgency of the COVID-19 pandemic. Demographic and clinical data were retrieved from medical records and through telephone interviews. Serum samples were collected from each patient between 15 February 2020 and 15 March 2020. Follow-up serum samples were collected between 7 June 2020 and 7 July 2020.

### Gold immunochromatographic assay

Serum IgM and IgG antibodies to SARS-CoV-2 were determined using a commercially available colloidal gold immunochromatographic assay kit (Nanjing Vazyme Medical Technology Co, Nanjing, China), which was approved by the Chinese National Medical Products Administration. Mammalian-cell-expressed recombinant antigens containing the N protein and the spike protein of SARS-CoV-2 were used to detect the anti-SARS-CoV-2 IgG and IgM antibodies. The sensitivities and specificities are 86.6% and 96.8% for IgM, 87.1% and 99.2% for IgG, respectively. The tests were performed according to the manufacturer’s instructions. Briefly, to detect IgG and IgM, 20 µL of serum and 60 µL of diluent were added to antigen-coated wells (test card). The test card was incubated at room temperature and the results were read after exactly 10 minutes. Every test card had a control line, and the results were only considered valid when the control line showed.

### Statistical analyses

For the descriptive analysis, continuous variables are presented as medians and interquartile ranges (IQRs), and categorical variables are presented as percentages. The chi-square test was used to test for the significance of differences between groups. A multivariable logistic regression analysis was performed to identify factors associated with a short duration (4–6 months) of anti-SARS-CoV-2 IgG antibody persistence. All statistical analyses were performed using SPSS Statistics, Version 26.0 (IBM Corp., Armonk, NY, USA). Two-sided *P*-values < 0.05 were considered statistically significant.

## Results

A total of 94 patients were included in the cohort, of whom 71 patients were diagnosed by polymerase chain reaction assays of nasal and/or pharyngeal specimens, and 23 were diagnosed on the basis of serum anti-SARS-CoV-2 antibody detection, symptoms compatible with COVID-19, and typical chest radiographic findings. Their median age was 65 years (IQR 58–69 years), and 48 (51.1%) were women. Fifty-two patients (55.3%) had comorbidities, including hypertension, diabetes, and coronary heart disease. Of the 94 patients, 49 (52.1%) had severe disease and 25 (26.6%) had been treated with systemic steroids.

All 94 patients underwent two IgG and IgM antibody tests. The first tests were conducted a median of 35 days (IQR: 29–39 days) after the onset of symptoms, and the first anti-SARS-CoV-2 IgG antibodies were all positive (Table 1). The second tests were conducted a median of 147.5 days (IQR 132–160 days) after the onset of symptoms. As shown in Table 2, 19 patients (21.8%) were IgG negative by 4–6 months after symptom onset.

**Table 1:** Results of the first anti-SARS-CoV-2 antibody tests among 94 participants following SARS-CoV-2 infection

	**IgG**		**IgM**	
**Time after symptom onset (days)**	No. samples tested	No. positive samples (%)	No. samples tested	No. positive samples (%)
**13–21**	5	5 (100.0)	5	4 (80.0)
**22–30**	22	22 (100.0)	22	15 (68.2)
**31–60**	67	67 (100.0)	67	47 (70.1)

*IgG* immunoglobulin G, *IgM* immunoglobulin M, *SARS-CoV-2* severe acute respiratory syndrome coronavirus 2

**Table 2:** Results of second anti-SARS-CoV-2 antibody tests among 94 participants following SARS-CoV-2 infection

	**IgG**		**IgM**	
**Time after symptom onset (days)**	No. samples tested	No. positive samples (%)	No. samples tested	No. positive samples (%)
**91–120**	7	7 (100.0)	7	1 (14.3)
**121–180**	87	68 (78.2)	87	5 (5.7)

*IgG* immunoglobulin G, *IgM* immunoglobulin M, *SARS-CoV-2* severe acute respiratory syndrome coronavirus 2

The trend in anti-SARS-CoV-2 IgM positivity differed from that of IgG. At 13–21 days after symptom onset, the IgM positivity rate was 80%. This rate decreased significantly to 5.7% at 4–6 months after symptom onset.

Of the 87 patients who had their second anti-SARS-CoV-2 IgG and IgM antibody tests 4–6 months after symptom onset, we performed a comparative analysis of clinical and demographic characteristics and observed no significant differences in age, sex, illness severity, comorbidities, or systemic steroid treatment between patients who remained IgG-seropositive and those who became seronegative over time. Multivariable logistic regression analysis, including age, sex, the severity of illness, comorbidities, and systemic steroid treatment, did not identify any factors significantly associated with a short duration of detectable IgG.

## Discussion

A previous study showed that the seroconversion of most COVID-19 patients occurs between 7 and 14 days after symptom onset [10]. However, the duration of anti-SARS-CoV-2 antibodies and risk factors associated with the duration are still uncertain. In our study, the first anti-SARS-CoV-2 IgG antibodies of 94 patients were all positive. However, about one fifth reverted to an IgG-negative status at 4–6 months after symptom onset. Furthermore, we did not identify any patient characteristics that were significantly associated with the persistence of IgG. Yang et al. [11] reported a rapid decline of anti-SARS-CoV-2 antibody levels in 34 patients with mild COVID-19 during an observation period of approximately 90 days. However, they also added that it is difficult to extrapolate beyond this time period because it is likely that the decay will decelerate. Gudbjartsson et al. analyzed serum samples from 1215 persons who recovered from SARS-CoV-2 infection and found that 91.9% remained seropositive within 4 months of SARS-CoV-2 infection [12]. In our study, the positivity rate of anti-SARS-CoV-2 IgG antibody was 100% at 4 months after symptom onset and then declined rapidly. This suggests that immunity to SARS-CoV-2 disappears 4–6 months after infection in some individuals, who may then become reinfected. To our knowledge, our study is the longest longitudinal anti-SARS-CoV-2 IgG and IgM follow-up reported to date.

A previous study found that higher antibody titers were independently associated with more severe disease [9]. Long et al. [13] showed that 40.0% of asymptomatic individuals and 12.9% of symptomatic individuals became IgG seronegative during the early convalescent phase (8 weeks after hospital discharge), and they suggested that asymptomatic individuals may develop a weaker immune response to SARS-CoV-2 infection. In our cohort, we found that neither severity of illness nor age, sex, comorbidities, and systemic steroid treatment were associated with a short duration of detectable IgG.

## Conclusions

Our data suggest that a substantial fraction of convalescents may exhibit a transient IgG response following SARS-CoV-2 infection and revert to a seronegative status within 4–6 months after symptom onset; the duration of this response is not associated with symptom severity. Furthermore, our findings raise concerns about the possibility of reinfection among previously exposed individuals. Thus, patients who have recovered from SARS-CoV-2 infection should also be vaccinated if their anti-SARS-CoV-2 IgG antibodies are undetectable.

## Data Availability

The data used to support the findings of this study are available from the corresponding author upon request.
